# Non-*Saccharomyces* as Biotools to Control the Production of Off-Flavors in Wines

**DOI:** 10.3390/molecules26154571

**Published:** 2021-07-28

**Authors:** Antonio Morata, Iris Loira, Carmen González, Carlos Escott

**Affiliations:** enotecUPM, Departamento de Química y Tecnología de Alimentos, Universidad Politécnica de Madrid, Avenida Puerta de Hierro 2, 28040 Madrid, Spain; iris.loira@upm.es (I.L.); carmen.gchamorro@upm.es (C.G.); carlos.escott@gmail.com (C.E.)

**Keywords:** wine, yeasts, non-*Saccharomyces*, off-smells, volatile acidity, ethylphenols, pyranoanthocyanins, pH control, bioprotection

## Abstract

Off-flavors produced by undesirable microbial spoilage are a major concern in wineries, as they affect wine quality. This situation is worse in warm areas affected by global warming because of the resulting higher pHs in wines. Natural biotechnologies can aid in effectively controlling these processes, while reducing the use of chemical preservatives such as SO_2_. Bioacidification reduces the development of spoilage yeasts and bacteria, but also increases the amount of molecular SO_2_, which allows for lower total levels. The use of non-*Saccharomyces* yeasts, such as *Lachancea thermotolerans*, results in effective acidification through the production of lactic acid from sugars. Furthermore, high lactic acid contents (>4 g/L) inhibit lactic acid bacteria and have some effect on *Brettanomyces*. Additionally, the use of yeasts with hydroxycinnamate decarboxylase (HCDC) activity can be useful to promote the fermentative formation of stable vinylphenolic pyranoanthocyanins, reducing the amount of ethylphenol precursors. This biotechnology increases the amount of stable pigments and simultaneously prevents the formation of high contents of ethylphenols, even when the wine is contaminated by *Brettanomyces*.

## 1. Introduction

Wine quality is strongly and negatively affected by some microbial metabolites with low sensory thresholds and negative olfactory impact, including reduced sulfur compounds [1,2], volatile acidity [3], ethylphenols [4,5], and acetaldehyde [6]. The sensory impact is quite variable because sensory thresholds can range from very low values (H_2_S, 1.6 μg/L) to very high concentrations (volatile acidity 0.3–0.6 g/L), so the range is about 1 million times (Table 1). This makes the analytical approach very specific and makes the use of sensitive and reproducible techniques based mainly on gas chromatography–mass spectrometry (GC-MS or GC-MS/MS) instruments essential for their determination [7]. These analytical methods often require specific sample preparations and concentrations: headspace (HS), dynamic headspace (DHS), solid-phase microextraction (SPME), and Twister [7].

The control of fermentative purity as well as the development of wild spoilage microorganisms in wines are related to pH and sulfur dioxide contents. In warm areas affected by global warming, pH values have been increasing in recent years, which are associated with higher alcoholic strength and intense phenolic ripening [12,13,14], especially in varieties that accumulate high potassium contents in berries [15,16]. A high pH produces wines that are more chemically and microbiologically unstable, and therefore are more susceptible to microbial spoilage, including off-flavor formation. Wine pH can range from 2.8 to 4.5, although most wines are in the 3 to 4 range. However, wines below 3.5 are very stable and usually less affected by microbial developments, while wines with pHs close to 4 are very risky as many spoilage bacteria and yeasts can easily develop in them during processing and especially during aging and storage.

**Figure 1:**
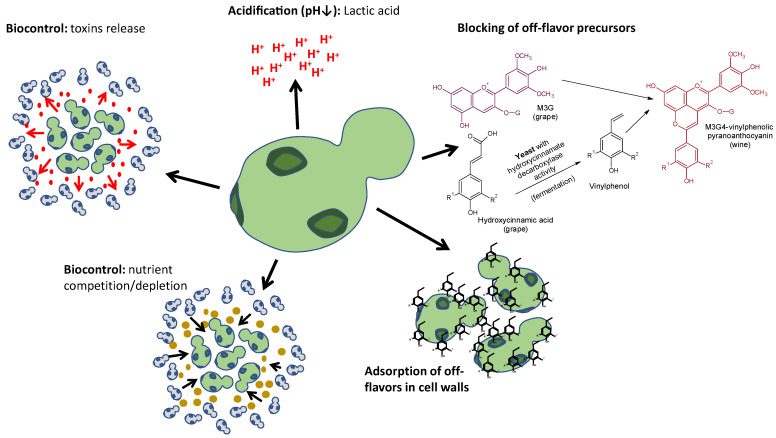
Summary of the mechanisms of biocontrol of off-flavor formation by yeasts. M3G: malvidin-3-*O*-glucoside; M3G4-vinylphenolic: malvidin-3-*O*-glucoside-4-vinylphenolic.

Yeast species can help in the biocontrol of off-flavor formation via bioprotection as a result of competition with or the elimination of wild spoilage microorganisms [17,18,19], by acidity production [20,21,22,23], by nutrient competition and depletion [24,25], by the depletion of off-flavor precursors [26], or by the adsorption of defective molecules on cell walls [27,28] (Figure 1).

This review is focused on the elimination of off-flavors by using non-*Saccharomyces* yeasts that are able to control pH by bioacidification or to decrease the concentration of precursors of molecules responsible for sensory defects.

## 2. Bioprotection

Bioprotection is a current concept, so its definition is still under discussion. However, it can be considered the active or passive use of some microorganisms to preserve foods and beverages and to exclude other spoilage microorganisms, thus avoiding the production of off-flavors, sensory alterations, or even the formation of toxic molecules. Bioprotection is a hot topic in enology and foods; several reviews have recently been published [29,30,31]. Bioprotection can be achieved by the production of molecules or metabolites with antimicrobial effects such as organic acids [32]; toxins such as killer factors [33,34,35]; deleterious chelates such as pulcherrimin [25,36]; glucanases [30,37]; ethanol produced in the fermentation of sugars; as well as by nutrient depletion [38] (Figure 1).

The application of bioprotective microorganisms can be scheduled at several stages of the winemaking process [31]. During the prefermentative stage or during prefermentative maceration, they can be applied directly on the grapes (harvesting machine) to control wild yeast [39,40], mold, and bacteria populations [19,41]. During fermentation, they can be applied to control the development of spoilage yeasts and bacteria and the oxidative processes, and frequently also to improve the sensory profile of wines by producing flavor compounds [18,42]. Finally, they can be applied after fermentation to protect and stabilize wines during barrel and bottle aging.

Bioprotection has been proposed as an effective biotool to reduce SO_2_ levels in wines [19,41]. The non-*Saccharomyces Torulaspora delbrueckii* (Td) and *Metschnikowia pulcherrima* (Mp) have been used to produce industrial fermentations without added sulfites. These bioprotective non-*Saccharomyces* may control some spoilage microorganisms and prevent chemical and enzymatic oxidation [41,43].

Regarding off-flavor formation, *Metschnikowia fructicola* has been successfully used to reduce the production of ethyl acetate by apiculate yeasts such as *Hanseniaspora uvarum* during cold soak [44].

## 3. Bioacidification by *Lachancea thermotolerans* (Lt)

Acidification and pH control are key tools in enology to preserve wine stability and prevent microbial spoilage. Tartaric acid, the strongest acid in grapes, is systematically used in many wines, particularly in warm areas, to improve chemical stability, enhance color and stabilize anthocyanins, increase the levels of active molecular SO_2_, and improve wine freshness [45]. Additionally, other acids such as malic, lactic, and citric acid can be used in enology, as can alternative physicochemical processes such as exchange resins and electrodialysis [45]. Acidification with up to 4 g/L tartaric acid is allowed in wines [9].

Bioacidification with *Saccharomyces cerevisiae* (Sc) through malic acid production has been previously studied. Some Sc strains can produce up to 1 g/L [46] when acidification occurs at the beginning of fermentation (days 2–6). However, the production at the highest level takes place in musts with low malic acid content and low acidity. Moreover, the effect on pH from increasing malic acid by 1 g/L is low and can be degraded by lactic acid bacteria, and thus malic acidification by Sc is not an effective biotechnology in winemaking. Other acids such as lactic, fumaric, and citric acids are also produced by Sc, but at low concentrations and with little impact on wine pH.

Lactic acid is also used for wine acidification, and the sensory effect is better in postfermentation acidifications. Even when often associated with dairy products, the sensory profile of lactic acid is fresh and citric [47]. Lactic acid is also authorized by the OIV for wine acidification. However, the use of lactic acid bioproduction by *Lachancea thermotolerans* is a natural and powerful biotool to control wine pH [20,21,22,23,48,49]. Some strains are capable of producing more than 16 g/L [21]. This amount is likely to be excessive in enological applications, and thus the use of strains with yields ranging from 5 to 8 g/L may be more appropriate [50,51,52]. With these conditions, it is easy to achieve pH reductions of 0.4–0.5 units [50,51,52], which is more effective than the usual acidification with tartaric acid. The production of L(+)-lactic acid [53] is done by the metabolization of sugars so that some reduction in alcoholic strength can be obtained, ranging from 0.2 [50] to 0.9% vol. [52]. Additionally, this strong reduction in pH favors higher levels of molecular SO_2_ under enological conditions (Table 2), which is very effective in controlling undesirable spoilage microorganisms and off-flavor production. The usual total SO_2_ contents (40–60 mg/L) at the typical high pH of grape juice in warm areas (3.7–4.0) can produce ineffective molecular SO_2_ contents (<0.5 mg/L) (Table 2). These conditions are suitable for the development of spoilage microorganisms that may increase the content of off-flavors or even allergenic or toxic molecules in wines, such as biogenic amines or ethyl carbamate, which can increase during wine aging. The same levels of total SO_2_ at pH 3.4–3.5 that can be obtained by Lt acidification during fermentation can easily produce molecular SO_2_ levels above 0.8 mg/L, resulting in a more protective effect and a safer situation (Table 2).

**Figure 2:**
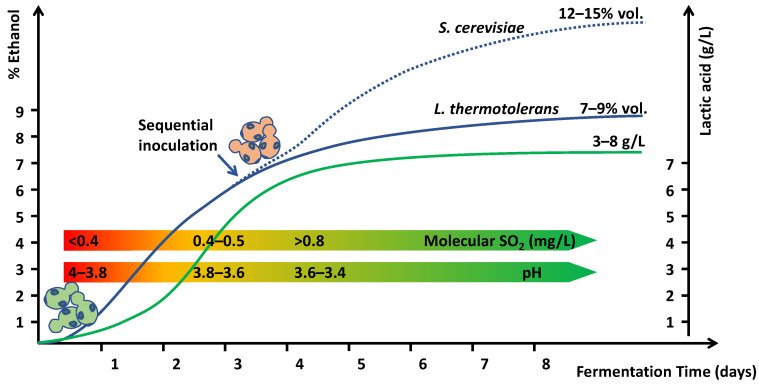
Sequential fermentations with *Lachancea thermotolerans* (green yeasts—bottom left) and *Saccharomyces cerevisiae* (pink yeasts—center). Effect on lactic acid production (green line), alcohol content (blue line), pH, and molecular SO_2_.

Therefore, Lt fermentations are a potent biotool to promote wine stability by reducing pH and increasing molecular SO_2_ levels. The use of Lt in sequential fermentations produces a significant reduction in pH and a concomitant effect on molecular SO_2_ (Figure 2). To produce complete fermentations without residual sugars, it is necessary to inoculate some non-*Saccharomyces* yeasts because most Lt strains have fermentative powers ranging from 7% to 9% *v*/*v* [20,23].

A typical stage in the stabilization of red wines is the need to carry out malolactic fermentation (MLF) and improve the sensory profile. However, MLF results in a reduction of pH, and in warm areas the sensory perception can be flat with a less crispy acidity sensation in the mouth. In these winemaking regions, it could be interesting to preserve malic acidity by inhibiting MLF and simultaneously lower the pH by producing lactic acid with Lt. It should be noted that lactic acid is a strong inhibitor of MLF at high doses, which occurs in many enzymatic processes and is known as product inhibition. It has been observed that lactic acid concentrations above 4 g/L produce a strong inhibition of MLF [32] and significantly decrease lactic acid bacteria populations. At lower values (2 g/L), a significant delay of MLF is observed [32]. Therefore, in addition to pH control by Lt acidification, effective inhibition of MLF can be obtained when lactic acid production is higher than 4 g/L. Furthermore, other malic-acid-preserving additives such as fumaric acid or chitosan can be used to control MLF [54]; the former is in the final stages of evaluation at the OIV [55] and the latter is also authorized for organic wines. Additionally, it has been observed that fumaric acid production can be increased by engineered Sc to more than 5 g/L [56]. Overproduction is done by overexpression of the RoPYC gene, so perhaps in some countries where the use of engineered yeasts is allowed, it can aid in inhibiting MLF along with Lt.

Another interesting application of Lt is the control of volatile acidity levels and most likely of ethyl acetate contents as well [57]. In addition, several authors have reported low volatile acidity contents (<0.5 g/L) in sequential fermentations with Lt [20,22,50,58], even in ternary fermentations with Lt and other non-*Saccharomyces*, such as *Metschnikowia pulcherrima*, *Torulaspora delbrueckii* or *Hanseniaspora vineae* [59], and ethyl acetate contents similar to Sc controls [50].

Lactic acid production and pH reduction by Lt also have concomitant effects on color due to the increased amounts of pyrylium cation in the wine, resulting in a hyperchromic effect and color protection [60,61].

Furthermore, Lt fermentations have shown preliminary positive effects on *Brettanomyces* control, likely due to acidification and the high contents of lactate [52].

## 4. Apiculate Yeasts and Volatile Acidity

Traditionally, apiculate yeasts (*Hanseniaspora*/*Kloeckera* species), usually involved in the early fermentation phases, have been considered overproducers of volatile acidity and ethyl acetate [62]. Pure culture fermentations of *Hanseniaspora uvarum* and *Kloeckera apiculata* have been reported to produce up to 0.98 and 1.5 g/L acetic acid, and 408 and 225 mg/L acetoin, respectively. In fact, they usually release high contents of acetate esters during fermentation; such is the case for the accumulation of ethyl acetate with concentrations between 450 and 760 mg/L. However, not all species behave in the same way and some of them, such as *Hanseniaspora vineae* (Hv), have shown a high ability to decrease volatile acidity in sequential fermentations with *Saccharomyces cerevisiae* compared to single Sc fermentations [63,64]. In triplicate fermentations of white wines, the Sc control produced 0.45 g/L acetic acid, but the sequential fermentation with Hv/Sc produced 0.36 g/L [65]. Additionally, Hv can produce significant amounts of floral and fruity acetate esters, benzenoids, and terpenes, improving the aroma profile of flat neutral varieties [63,64,66,67]. Furthermore, Hv is better adapted to the fermentation process and it is possible to select strains capable of reaching 10% ethanol [68]. The use of other *Hanseniaspora* species such as *H. opuntiae* fermenting Cabernet Sauvignon red grapes has also shown low volatile acidity values together with positive fruity and floral profiles [69,70].

## 5. Biocompatibility

The use of non-*Saccharomyces* in ternary cultures (two non-*Saccharomyces* species and one *Saccharomyces* species) in sequential or mixed fermentations has several advantages in terms of aroma improvement, control of spoilage microorganisms, and depletion of off-flavors; however, it is very important to ensure the biocompatibility of the strains used. When Lt has been used in co-inoculation with Hv, Td, and Mp, the latter has shown very good compatibility with Lt, reaching even higher levels of acidification than using only Lt in sequential fermentation with Sc [59]. However, the simultaneous use of Lt and Td decreased acidification, and the pH was higher compared to Lt alone, but lower than in the Sc control. The Lt and Hv strains showed the worst effectiveness on pH reduction, despite our high expectations of the complementary effect of both yeasts on acidity and aroma. This may be caused by the extra consumption of thiamine and pantothenate by Hv and the potential depletion of these important micronutrients, particularly of thiamine. The genes for thiamine biosynthesis in Hv and other *Hanseniaspora* species have not yet been identified, and this may explain the increased requirements of this vitamin in Hv fermentations [68,71,72]. Thiamine consumption and depletion may affect the development of other non-*Saccharomyces* species when used in co-fermentation.

## 6. Depletion of Off-Flavor Precursors

**Figure 3:**
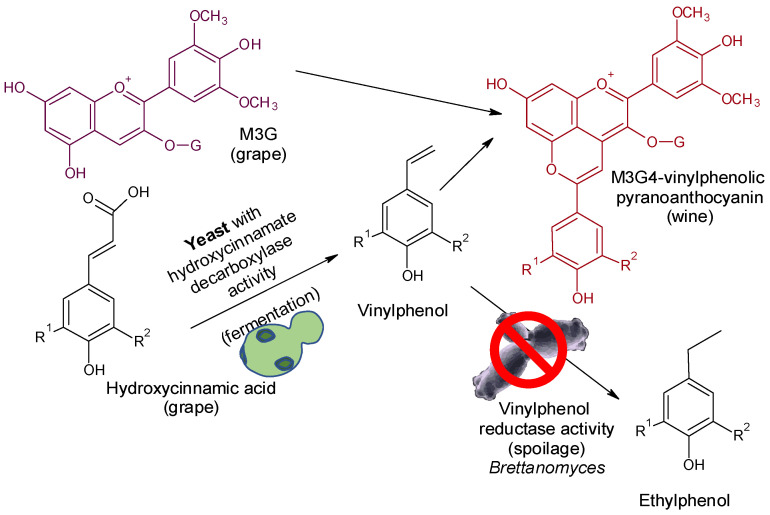
The depletion of ethylphenol precursors by the metabolization of hydroxycinnamic acids to vinylphenols and the blocking of this by reaction with grape anthocyanins to form vinylphenolic pyranoanthocyanins.

The production of some off-flavors that are extremely deleterious to wine quality, such as ethylphenols (EPs) [4,5], is highly dependent on precursor content. EPs are formed from hydroxycinnamic acids (HCAs) or their tartaric esters (TE-HCAs) by the sequential activities of hydroxycinnamate decarboxylase (HCDC) and vinylphenol reductase (VPR) from *Brettanomyces*/*Dekkera* [4,10]. Several technologies have been proposed to control *Brettanomyces* in wines, including emerging non-thermal technologies, some additives, and biotechnologies [4,73]. Many Sc strains express HCDC activity, but VPR activity has not been described in this species. Some Sc strains express HCDC activity with high intensity with the ability to transform most hydroxycinnamic acids into vinylphenols (VPs) (Figure 3). Moreover, it has been observed that these VPs can spontaneously react with grape anthocyanins to form vinylphenolic pyranoanthocyanins (VPAs) [74,75], which are stable pigments under enological conditions, as they are less affected by pH, oxidations, and sulfur dioxide bleaching than grape anthocyanins [61,76,77,78,79]. The use of Sc with an appropriate expression of HCDC activity is a powerful and natural biotool to favor the enzymatic metabolization of HCAs to VPs and the subsequent reaction with grape anthocyanins to form VPAs. This biological process blocks the EP precursors into stable VPAs, which are positive in terms of color stability, but also preserves the wines from the effect of *Brettanomyces*/*Dekkera*. When 10 commercial yeasts with verified HCDC activity were used to ferment red musts and subsequently contaminated with *Brettanomyces*, the 4EP content ranged from 22 to 498 µg/L, which is below or close to the sensory threshold of 4EP in wines [26]. However, in the control yeast (without HCDC activity), the 4EP content was 1150 µg/L, more than twice the sensory threshold [26]. Furthermore, most of the HCAs in grapes are found as tartaric esters (TE-HCAs); that is, caftaric, coutaric, and fertaric acids are reservoirs of HCAs that can be released by acid hydrolysis during aging. The use of cinnamyl esterase enzymes during fermentation can release the HCAs which, using Sc with HCDC activity, can be transformed into VPs and subsequently into VPAs by condensation with grape anthocyanins [26]. In addition, the use of some non-*Saccharomyces* yeast strains such as *Torulaspora delbrueckii* or *Metschnikowia pulcherrima* can enhance the formation of VPAs [60,80].

## 7. Increasing the Implantation of Non-*Saccharomyces* as Bioprotective Tools Using Emerging Non-Thermal Technologies

To achieve a good effectiveness with non-*Saccharomyces* yeasts in off-flavor control through bioprotection, acidification, and improved sulfur dioxide efficiency or precursor depletion, it is necessary to reach a good implantation of the intended species. One of the main drawbacks of non-*Saccharomyces* yeasts is the low fermentative power (<10% vol. and in many species <4% vol.) and the low fermentative yield, which generally results in poor implantation compared to Sc. To improve this, the use of non-thermal emerging technologies is compelling and effective because they can effectively eliminate wild yeasts, but also have little impact on the sensory components of the musts, that is, the aroma, pigments, and flavors [73,81,82,83].

The emerging non-thermal technologies include pressurization technologies (high hydrostatic pressure, HHP [84], and ultra-high-pressure homogenization, UHPH [85]), pulsed electric fields (PEFs) [86], pulsed light (PL) [87], irradiation (βI) [88], cold plasma (CD) [89], and ultrasound (US) [90]. All of them except US have demonstrated a good capacity to inactivate wild yeasts and even bacteria in grapes and musts, and preserve sensory and nutritional quality. HHP can produce reductions in wild yeast populations of more than 4-log [91,92], but residual bacterial counts can remain. UHPH is capable of producing sterilization with the elimination of yeast, bacteria, and even spores, depending on the in-valve temperature [85,93]. Pulsed technologies (i.e., PEFs and PL) have shown an inactivation capacity around or above 2-log for wild yeasts in grapes [94,95,96]. The antimicrobial performance of PEFs can be greatly enhanced in combination with mild temperatures (50 °C) [97].

The inactivation of wild yeasts by emerging non-thermal technologies in grapes or grape must is a useful technology to facilitate the implantation of non-*Saccharomyces* starters that can be used to control off-flavor formation. Several non-thermal technologies have shown high efficiency in increasing the implantation of non-*Saccharomyces* yeasts, such as HHP [92] and PEFs [95]. The high effectiveness of UHPH also makes it a leading technology not only for improving yeast implantation, but also for reducing SO_2_ levels due to its ability to inactivate oxidative enzymes [85].

In addition, when used on grapes, several of these non-thermal technologies are able to increase the extraction of phenolic compounds, thus improving the tannin and anthocyanin content of the wine. An increase in anthocyanin extraction ranging from 23% to 63% by HHP [98,99], 21% to 29% by PEFs [100], and the same contents but with reductions of more than 50% in maceration time by US [101] have been published.

## 8. Conclusions

The use of non-*Saccharomyces* in wine fermentation is a verified biotechnology to improve the sensory profile, and is also a powerful biotool to control off-flavor formation by the biocontrol of spoilage microorganisms, by pH control and the improvement of molecular SO_2_ contents by acidification, and by the depletion of precursors, among many other potential future possibilities.

## Figures and Tables

**Table 1:** Wine off-flavors produced by microbial metabolites.

Compound	Sensory Threshold	Off-Flavor Concentration ^1^	Descriptor	Reference
H_2_S	1.6 μg/L	>1.6 μg/L	Rotten eggs/putrefaction	[2]
Volatile acidity	0.3–0.6 g/L	>0.8 g/L legal limit 1.2 g/L	Vinegar	[7,8,9]
Ethyl acetate	12 mg/L	>150 mg/L	Glue, solvent	[7]
4-Ethylphenol	230 μg/L	>425 μg/L	Phenolic, stable, leather, horse sweat	[5,10,11]
Acetaldehyde	100–125 mg/L	>125 mg/L	Fruity, rotten apples, nut-like, sherry	[6]

^1^ The sensory threshold and off-flavor perception can be variable depending on the structure, composition, and sensory buffering effect of the wine.

**Table 2:** Bioacidification by *Lachancea thermotolerans* strain L31 and effect on pH and molecular SO_2_ in several fermentations with red and white varieties in different Spanish regions. Colors indicate the effectiveness of molecular SO_2_ from unideal (red) or less than optimal (yellow) to optimal (green) depending on the increase in acidity.

Variety (Region)	Inoculation	Lactic Acid (g/L) and Initial→Final pH	Effect of Acidity on the Molecular SO_2_ (mg/L) *	Reference
Tempranillo (Ribera del Duero)	Sequential with *S. cerevisiae*	0.91→6.60 g/L 3.90→3.63	0.42→0.77	[50]
Tempranillo (Ribera del Duero)	Mixed with *O. oeni* and sequential with *S. cerevisiae*	0.91→7.50 g/L 3.90→3.31	0.42→1.56	[50]
Tempranillo (Mancha)	Sequential with *S. cerevisiae*	3.8→3.4	0.50→1.22	Unpublished
Albariño (Rias Baixas-O Rosal)	Sequential with *S. cerevisiae*	0.05→2.7 g/L 3.12→2.85	2.07→3.63	[50]
Airén (La Mancha)	Sequential with *S. cerevisiae*	0.05→4.20 g/L 3.75→3.35	0.51→1.25	[51]

* Comparison for a total content of SO_2_ of 50 mg/L.

## Data Availability

The review study did not report any data.

## Sample Availability

Samples of the compounds ... are available from the authors.
